# Sustainable Production of Microbial Isoprenoid Derived Advanced Biojet Fuels Using Different Generation Feedstocks: A Review

**DOI:** 10.3389/fbioe.2020.599560

**Published:** 2020-10-30

**Authors:** Laura Ellen Walls, Leonardo Rios-Solis

**Affiliations:** ^1^Institute for Bioengineering, School of Engineering, The University of Edinburgh, Edinburgh, United Kingdom; ^2^Centre for Synthetic and Systems Biology (SynthSys), The University of Edinburgh, Edinburgh, United Kingdom

**Keywords:** advanced biojet fuel, isoprenoid, monoterpene, sesquiterpene, lignocellulosic biomass, consolidated bioprocessing

## Abstract

As the fastest mode of transport, the aircraft is a major driver for globalization and economic growth. The development of alternative advanced liquid fuels is critical to sustainable development within the sector. Such fuels should be compatible with existing infrastructure and derived from second generation feedstocks to avoid competition with food markets. With properties similar to petroleum based fuels, isoprenoid derived compounds such as limonene, bisabolane, farnesane, and pinene dimers are of increasing interest as “drop-in” replacement jet fuels. In this review potential isoprenoid derived jet fuels and progress toward their microbial production was discussed in detail. Although substantial advancements have been achieved, the use of first generation feedstocks remains ubiquitous. Lignocellulosic biomass is the most abundant raw material available for biofuel production, however, technological constraints associated with its pretreatment and saccharification hinder its economic feasibility for low-value commodity production. Non-conventional microbes with novel characteristics including cellulolytic bacteria and fungi capable of highly efficient lignocellulose degradation and xylose fermenting oleaginous yeast with enhanced lignin-associated inhibitor tolerance were investigated as alternatives to traditional model hosts. Finally, innovative bioprocessing methods including consolidated bioprocessing and sequential bioreactor approaches, with potential to capitalize on such unique natural capabilities were considered.

## Introduction

Transportation is critical to sustainable development and global economic growth. However, despite efforts to develop alternative fuels, reliance on fossil fuels is ongoing. In 2016, just 7.1% of all transport fuel consumed in the European Union was from a renewable source and the transport sector contributed to 27% of greenhouse gas emissions. Similarly, in the United States, biofuels currently make up just 5% of transportation fuel consumption (U.S. Energy Information Administration, 2019). Globally, bioethanol is the most widely used biofuel due to its high level natural production by microbes (Mohd Azhar et al., 2017). However, the majority is currently derived from corn or sugarcane feed stocks, which utilize vast areas of agricultural land and threaten food security. Biodiesel, produced through the transesterification of vegetable oils, is the predominant renewable transport fuel in Europe. Such biodiesel is also a first generation biofuel, requiring agricultural land that could otherwise be used to grow food crops. There is an increasing interest in low emission electric vehicles due to their reduced contribution to urban air pollution with a number of countries offering incentives for electric vehicles (UK Government (Department for Transport), 2020, 2018; Sherlock, 2019). In addition, companies including Rolls Royce, Airbus, Eviation and magniX are driving innovation to develop all electric aircrafts. Recently Eviation (2019) announced the development of Alice, a nine passenger aircraft with a range of 650 miles. Although this represents a major technological breakthrough, the limited capacity of current battery technology is a major bottleneck. The state-of-the-art lithium ion batteries used to power Alice have an energy density of 248 Wh/kg (KOKAM, 2019), which is just 2% of that of Jet A (Table 1), substantial advancements in lightweight energy storage technology are therefore necessary. As a result, for aviation, shipping and heavy good vehicles, reliance on liquid fuels is likely to continue for some time (The Royal Academy of Engineering, 2017).

**TABLE 1:** Properties of standard jet fuels.

Fuel	Property						
	Energy density (MJ/kg)	Freezing point (°C)	Flash point (°C)	Viscosity (mm^2^/s)	Specific gravity	Chemical formula	References
Jet A	>42.8	< −47	>38	<8 (−20°C)	0.775–0.840	C9–C16	ASTM International (2020a)
Jet A-1	>42.8	< −40	>38	<8 (−20°C)	0.775–0.840	C9–C16	ASTM International (2020a)
JP-10	42.1	−79	53	<9 (−18°C)	0.94	C10	Meylemans et al. (2012), Coordinating Research Council Inc (1983)
Diesel 1-D	42–46	–	>38	1.3–2.4 (40°C)	0.85	C8–C25	Speight (2011), ASTM International (2020b)

The aircraft remains the fastest and most convenient means of transporting people and goods. As the aviation industry facilitates global business, international trade and tourism, it is a critical component of the global economy. The sector is experiencing immense growth and annual air passengers are expected to almost double to 7.8 billion by 2036. As air transport improves access to food, medication and educational resources, it is vital for the implementation of many of the UN’s 17 Sustainable Development Goals. However, if the environmental cost is to be eliminated, the introduction of sustainable alternatives to petroleum based aviation fuels is critical. This demand for sustainable advanced biofuels is widely recognized by national governments (UK Government (Department for Transport), 2018; Governement of India (Ministry of New and Renewable Energy), 2019), influential multinational organizations (United Nations, 2015; The European Parliament and of the Council of the European Union, 2018) and global oil and gas companies (ExxonMobil, 2018; Royal Dutch and Shell, 2019). The European Parliament and of the Council of the European Union (2018) recently introduced stringent renewable energy targets, requiring at least 32% of Europe’s energy be derived from a renewable source by 2030. In addition, first generation biofuel production must be restricted to a sustainable level of just 3.8%. The international air transport association (IATA) is a trade association with 290 airline members, representing 82% of the world’s air traffic. They have also set a number of ambitious targets for their member organizations, including a cap on net CO_2_ emissions from 2020 to promote carbon neutral growth and a 50% reduction in net CO_2_ emissions by 2050 compared to 2005 levels. The implementation of sustainable aviation fuels is imperative to the achievement of such goals. To be compatible with existing infrastructure, such fuels should have similar properties to traditional fuels (Table 1). Although similar to those used in diesel engines, a lower freezing point is paramount for aviation fuels to withstand the extreme cold.

**FIGURE 1:**
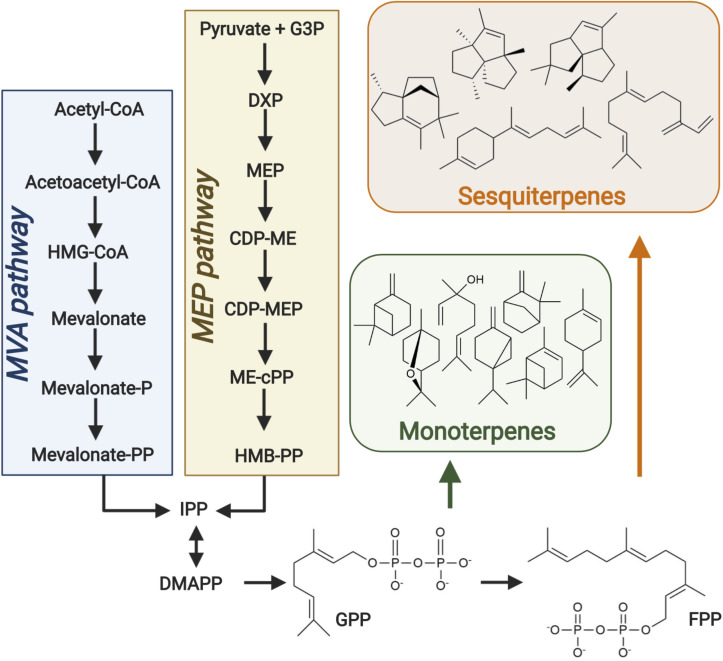
Isoprenoid biosynthesis. The universal monoterpene and sesquiterpene precursors geranyl diphosphate (GPP) and farnesyl diphosphate (FPP), respectively, are produced via both the mevalonate (MVA) and MEP pathways in plants. Terpene synthases then convert such precursors into a wide range of monoterpene and sesquiterpene compounds. Figure created with BioRender.com.

The potential of natural medium chain length isoprenoid and fatty acid derived hydrocarbons to meet such requirements is of growing research interest (Lee et al., 2008; Jiménez-Díaz et al., 2017). Isoprenoids are the largest and most diverse class of naturally occurring organic compounds. They have a vast range of applications in the pharmaceutical, fragrance and fuel industries and have enriched the everyday lives of humans for millennia (Peralta-Yahya et al., 2011; George et al., 2015). Isoprenoid research predominantly focused on medicinal applications such as the construction of microbial pathways for the ground-breaking antimalarial (Paddon and Keasling, 2014) and anticancer drugs (Wong et al., 2018; Nowrouzi et al., 2020; Walls et al., 2020). However, the aforementioned introduction of stringent greenhouse gas emission reduction targets in recent years has increased interest in their potential as advanced biofuels (Peralta-Yahya et al., 2012; George et al., 2015; Wong et al., 2017). Isoprenoids are formed through the condensation of two or more isoprene units and are classified based on the number of units they contain (Srivastava, 2002). Many isoprenoids have great potential as high-density fuels due to their compact cyclic structures, especially those in which ring strain contributes to a high heat of combustion. The physical properties and high energy contents of many isoprenoids (Peralta-Yahya et al., 2012) are synonymous to those of petroleum-based fuels (Table 1). Monoterpene (C_10_) and sesquiterpene (C_15_) compounds have carbon chain lengths in the range of conventional jet fuels (Table 1) and are therefore of particular interest as potential replacements. Such compounds are derived from geranyl diphosphate and farnesyl diphosphate, respectively, products of the mevalonate (MVA) and MEP pathways in plants (Figure 1).

Low quantities of isoprenoids typically accumulate in their natural hosts. As a result, the direct extraction and isolation of the compounds is low yielding and therefore cannot be employed for large scale production. In addition, slow plant growth and susceptibility to seasonal variations further limit extraction from plant sources (Chang and Keasling, 2006; Tippmann et al., 2013). Chemical synthesis presents an alternative method for isoprenoid production. However, the complex stereochemistry of isoprenoids complicates enantioselective synthesis and typically results in high costs and undesirable yields (Immethun et al., 2013; Tippmann et al., 2013). In addition, the hazardous solvents required for chemical synthesis pose serious health and safety as well as environmental risks (Tippmann et al., 2013).

The development of strategically designed microbial cell factories for the heterologous biosynthesis of isoprenoids from low cost, renewable feedstocks has the potential to provide a sustainable solution. Thanks to advancements in synthetic and systems biology technology, progress toward the construction of such cell factories has accelerated recently and microbial production of a wide range of potential isoprenoid biojet fuel feedstocks has now been demonstrated at laboratory scale (Peralta-Yahya et al., 2011; Brennan et al., 2015; Mendez-Perez et al., 2017; Liu et al., 2018, Liu Y. et. al., 2019). However, the development and scale up of economical bioprocesses for such cell factories at industrial scale remains a monumental challenge, hindering the realization of these wide-ranging benefits. One of the greatest challenges in the development of effective bioprocesses for the isoprenoid candidates, is the low value of the respective advanced biojet fuel products. In order to be economically viable at commercial scale, yields of the potential jet fuels must be substantially greater than for higher value products such as pharmaceuticals. Cell factories for biojet fuels are therefore likely to require more extensive and complex metabolic engineering (Meadows et al., 2016). In addition, the use of first-generation feedstocks, which are inherently favored by most microbial hosts, is unlikely to be feasible due to their high cost and competition with food markets. Second generation feedstocks such, which are derived from non-edible biomass sources such as lignocellulosic biomass, is the most abundant feedstock available for microbial biofuel production. As a low cost, renewable carbon source, lignocellulosic biomass is a highly attractive carbon source, however, expensive pretreatment requirements and the presence of inhibitory compounds has complicated its application to date. Novel third and fourth generation feedstocks are gaining increasing interest as feedstocks for microbial isoprenoid jet fuel production. Such generation fuels are derived from phototrophic microorganisms such as algae and cyanobacteria and therefore provide the additional environmental benefit of CO_2_ sequestration. However, as they are limited by the rate of photosynthesis, ensuring sufficient yields for commercial scale production is problematic.

In this review potential isoprenoid derived biojet fuel candidates and the challenges associated with the development of sustainable bioprocesses for their industrial-scale production will be discussed. Key bottlenecks including microbial host selection, feedstock selection and pretreatment requirements will be considered along with progress toward their alleviation. Finally, recent progress toward consolidated bioprocessing (CBP) will be discussed along with alternative sequential bioreactor approaches.

## Isoprenoid Aviation Fuel Candidates

### Monoterpene Biojet Fuels

Monoterpene are formed of two isoprene units and may be either acyclic or cyclic, however, cyclic monoterpenes are more abundant in nature and have found more industrial applications (Kang and Lee, 2016). The C_10_ hydrocarbon chain length of monoterpenes is in the desirable range of traditional jet fuels (C_8_–C_16_; Peralta-Yahya et al., 2012). The monocyclic monoterpene, limonene, is a promising feedstock for the biofuel industry. It is found naturally in the peel of citrus fruits and exists as two enantiomers, D-limonene which has a characteristic orange fragrance and L-limonene which has a turpentine like odor. D-limonene is a by-product of the citrus juice industry (Jongedijk et al., 2016) and has applications in the fragrance industry within perfumes and as a solvent in cleaning products. It is also a useful feedstock in the food and pharmaceutical industries (Sun, 2007). In contrast, L-limonene is predominantly used as a precursor for the biosynthesis of *S*-menthol, a major component of mint (Lin et al., 2017). Interest in limonene is growing as a potential bio-jet fuel candidate (Chuck and Donnelly, 2014; Zargar et al., 2017). The distillation profile of limonene is similar to that of the aviation fuel jet A-1 in blends up to 20% limonene. As a result, its blend with jet fuel would have minimal deleterious effects upon combustion quality and efficiency (Chuck and Donnelly, 2014). Limonene was found to be an excellent substitute for aviation kerosene and could potentially be used as a drop-in replacement without the need for chemical upgrading (Chuck and Donnelly, 2014). Its lower viscosity could also provide additional benefits such as increased atomization and pumping (Chuck and Donnelly, 2014).

**FIGURE 2:**
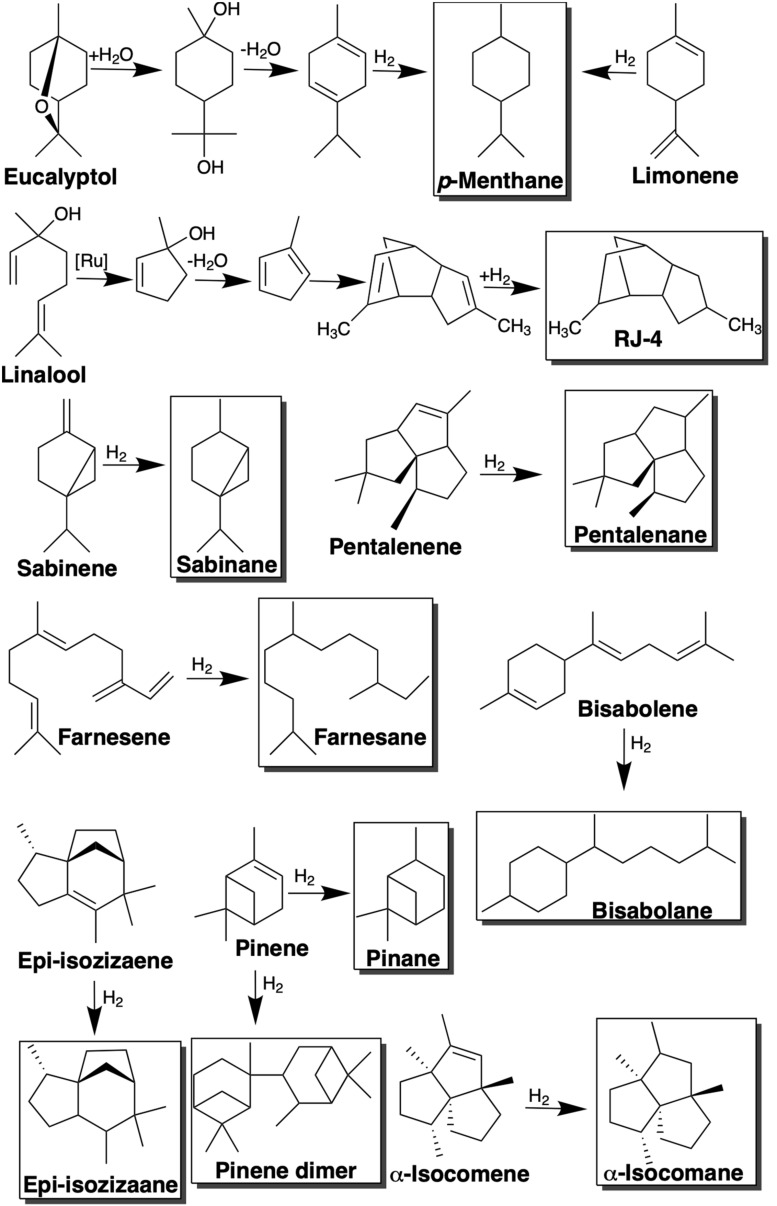
Conversion of monoterpene and sesquiterpene compounds into jet fuel blendstocks via hydrogenation, dimerization, or a combination of isomerization, dehydration, dimerization, and hydrogenation steps.

The oxygenated monoterpenoid, eucalyptol (1,8-cineole), is native to a number of species of the *Eucalyptus* genus and is widely used across the pharmaceutical, fragrance and flavoring industries (Mendez-Perez et al., 2017). More recently is has been shown to be a useful intermediate in the synthesis of *p*-cymene an important component of the biojet fuel blend AMJ-700t (10% cymene, 50% limonene, and 40% farnesene) (Brennan et al., 2015; Mendez-Perez et al., 2017). After a single hydrogenation step AMJ-700t can be converted into AMJ-700, the biojet fuel produced by Amyris, which successfully fueled an Azul Brazilian Airlines demonstration flight in 2012 (Brennan et al., 2015). Both eucalyptol and limonene can be chemically upgraded to the potential jet fuel blendstock, *p-*menthane, which has a high energy density and desirable low freeze point (Table 2). In the case of limonene this involves a single hydrogenation step, while eucalyptol must undergo ring opening, dehydration and hydrogenation to yield *p-*menthane (Figure 2; Baral et al., 2019). This reliance on a series of reactions for the conversion of eucalyptol has resulted in poor selectivity toward *p*-menthane (Yang et al., 2017). In order to tackle this, Yang et al. (2017), developed a “one-pot” biphasic tandem catalysis process to consolidate the three steps into a single unit operation for improved efficiency and selectivity. The resulting process achieved >99% conversion of eucalyptol to *p*-menthane at 120°C and 10 bar in just 1 h.

**TABLE 2:** Properties of potential isoprenoid derived biojet fuels.

Fuel	Property					
	Energy density (MJ/kg)	Freezing point (°C)	Flash point (°C)	Viscosity (mm^2^/s)	Specific gravity	References
Farnesene	∼44	−90	95+	14.28 (−20°C)	0.81	Chuck and Donnelly (2014)
Farnesane	43.39	< −100	96	2.35 (40°C)	0.77	Soriano et al. (2017); Pires et al. (2018)
Bisabolane	43.76	< −81	111	2.91	0.82	Peralta-Yahya et al. (2011)Baral et al. (2019)
Limonene	45	−74	50	∼2.5 (−20°C)	∼0.87	Chuck and Donnelly (2014)
*p-*Menthane	43.41	< −70	43	−	0.804	Ryder (2009); Baral et al. (2019)
Epi-isozizaane	42.584	–	–	–	0.929	Liu et al. (2018)
pentalenane	42.609	–	–	–	–	Liu et al. (2018)
α-isocomane	42.783	–	–	–	–	Liu et al. (2018)
Hydrogenated cedarwood oil	42.67	< −80	–	54 (−20°C)	0.917	Harrison and Harvey (2017)
AMJ-700	–	< −71	50	<10 (−20°C)	0.796	Ryder (2009)
RJ-4	42.45	−46	78	–	0.94	Osmont et al. (2006); Martel (1987)
α-pinane	43.14	−53	40	11.23 (−40°C)	0.86	Woodroffe and Harvey (2020)
Sabinane	43.25	–	–	4.809 (−40°C)	0.810	Woodroffe and Harvey (2020)
Camphene dimer	42.063	−54	–	34.96 (40°C)	0.941	Meylemans et al. (2012)
α-pinene dimer	42.047	−52	–	34.68 (40°C)	0.935	Meylemans et al. (2012)
β-pinene dimer	42.118	–	–	35.05 (40°C)	0.938	Meylemans et al. (2012)
Limonene dimer	41.906	−78	–	25.86 (40°C)	0.914	Meylemans et al. (2012)

The acyclic monoterpene, linalool, is another industrially important fragrance chemical, which has also been used as a precursor of the high-density fuel, RJ-4, used in missiles and as a component of jet fuel (Mendez-Perez et al., 2017). Through a series of isomerization, dehydrogenation, dimerization and hydrogenation steps, linalool can be chemically upgraded to RJ-4 as shown in Figure 2. As linalool requires more extensive chemical upgrading to yield RJ-4, the hydrogenation step accounts for a significantly greater proportion of the production costs (∼12%) than for other terpene hydrogenations such as limonene to *p-*menthane (Baral et al., 2019).

Bicyclic monoterpenes with potential biojet fuel applications include pinene, camphene and sabinene. Pinene is found naturally in the oils of numerous species of coniferous tree and exists as two isomers, α-pinene and β-pinene which are the major constituents of turpentine. The chemical dimerization of β-pinene yields a fuel with a very high volumetric energy, comparable with that of the super-dense military aviation fuel JP-10 (Harvey et al., 2010; Sarria et al., 2014). The strained ring structure of pinenes results in a higher heating value than linear molecules of a similar molecular weight. In addition, the dimerization of pinenes can substantially increase their fuel density. The boiling points of pinene dimers are in the range of 200–350°C making them suitable for diesel and jet fuels (Jung et al., 2016). Pinene trimers, on the other hand, are not suitable for biofuel applications due to their high viscosity and high boiling temperatures. The exocyclic double bond of the β-pinene isomer is preferable for dimerization as it provides an increased reactivity compared α-pinene. The conversion of α-pinene typically results in isomers with lower dimer and oligomer yields than β-pinene (Jung et al., 2016). Although, pinene dimerization can be achieved using a range of homogeneous or heterogeneous catalysts, Jung et al. (2016), observed higher β-pinene conversions with heterogeneous catalysts. Dimerization of camphene, another bicyclic monoterpene common to conifers, yields a fuel with similar properties to JP-10 (Meylemans et al., 2012).

Sabinene is a bicyclic monoterpene which has been isolated from a variety of plants such as holm oak. Its high energy density, low flash point and low freezing point makes it another promising advanced biojet fuel feedstock candidate (Wu et al., 2020). Hydrogenation of sabinene, yields sabinane, a promising jet fuel blendstock due to its high energy density and low viscosity (Table 2).

### Sesquiterpene Biojet Fuels

Sesquiterpenes are of growing research interest as potential advanced biojet fuel substitutes as a result of their high energy density and comparable cetane numbers to traditional jet fuels (Liu et al., 2018). Made up of three isoprene units, sesquiterpenes are C15 hydrocarbons and hence also fall in the desirable carbon chain length range of jet fuels (C_8_–C_16_; Peralta-Yahya et al., 2012). Since its successful commercial scale microbial production by Amyris, farnesane has become a particularly interesting jet fuel blendstock candidate (Oßwald et al., 2017). It is formed via the hydrogenation of farnesene, a naturally occurring sesquiterpene found in the coating of apples. The density and viscosity of farnesane are similar to those of diesel fuel (Table 1) and with a higher cetane number it displays superior combustion properties (Millo et al., 2014). Farnesane was recently approved for use in commercial jet fuels in blends up to 10% (ASTM International, 2020a). The viscosity of farnesene at −20°C is 14.28 mm^2^/s, however, above the upper limit of 8 mm^2^/s for standard jet fuel (ASTM International, 2020a). As a result, it is likely to require blending with other fuels for use in existing infrastructure. Despite this, farnesene can be successfully blended with other isoprenoid compounds to achieve the desired properties. For example, AMJ-700, which is the hydrogenated form of a mixture of farnesene, limonene and cymene as described in section “Monoterpene Biojet Fuels,” has a viscosity of <10 mm^2^/s at −20°C (Ryder, 2009) and meets the specifications of Jet A (Table 1; ASTM International, 2020a).

Bisabolene is a sesquiterpene found in a number of plant and fungal species. Its hydrogenated form, bisabolane, is another promising jet fuel candidate, its structure combines the combustion properties of a linear alkane with the increased energy density of a cyclic hydrocarbon giving it a comparable energy density to Jet-A fuel (Peralta-Yahya et al., 2011). However, hydrogenation required high pressures of around 82.7 bar and yielded a mixture of geometric isomers (Peralta-Yahya et al., 2011). In addition, as like farnesane, the viscosity of bisabolene is relatively high at ∼17 mm^2^/s at −20°C (Harvey, 2018), the compound would likely need to be blended with lower viscosity fuels for use in jet engines.

As the net heat of combustion of a fuel is highly dependent on its density, multicyclic sesquiterpenes are likely to be particularly suitable as high energy density fuels (Harrison and Harvey, 2017). Cederwood oil, derived from Redcedar trees, is comprised of the tricyclic sesquiterpenes α-cedrene, β-cedrene, thujopsene and the sesquiterpene alcohol, cedrol (Harrison and Harvey, 2017). Hydrogenated cedarwood oil has a high energy density of 42.67 MJ/kg and a desirable freeze point of < −80°C (Harrison and Harvey, 2017), however, its viscosity is considerably higher than the upper limits for standard jet fuel (ASTM International, 2020a) at 54 mm^2^/s at −20°C, which may hinder its performance as a drop in replacement. Liu et al. (2018) recently investigated three alternative novel tricyclic sesquiterpenes, epi-isozizaene, pentalenene and α-isocomene as potential jet fuel candidates. With predicted specific energies of 42.58, 42.61, and 42.78 MJ/kg, respectively, following hydrogenation, all three potential sesquiterpene fuels were envisaged to perform similarly to jet fuel A1 (42.8 MJ/kg). However, as successful hydrogenation of these sesquiterpenes is yet to be reported in literature, the potential yields and selectivity’s toward the saturated compound of interest are not known.

## Microbial Production of Isoprenoid Jet Fuel Candidates From First Generation Feedstocks

The chemical and physical properties (Table 2) of the monoterpene and sesquiterpene compounds discussed in section “Isoprenoid Aviation Fuel Candidates” are comparable to those of standard jet fuel (Table 1) rendering them inherently suitable for advanced biojet fuel applications. Although such compounds are found in nature, they are typically present in minute quantities in their natural host. Research has therefore focussed on the development of metabolically engineered microbial cell factories for the overproduction of mono and sesquiterpenes for biojet fuel applications. As a result of recent advancements in synthetic and systems biology, the construction and optimization of heterologous pathways in wide ranging microbial hosts is becoming increasingly straightforward. Consequently, substantial progress toward the development of effective cell factories for the production of isoprenoid derived biojet fuel candidates has been achieved in recent times. In this section significant examples of first generation, microbial isoprenoid biofuels will be discussed.

### E. coli

*Escherichia coli* has been successfully engineered to produce a number of isoprenoid jet fuel precursors (Peralta-Yahya et al., 2011; Mendez-Perez et al., 2017; You et al., 2017). Although this species is capable of synthesizing the critical isoprenoid building blocks, IPP and DMAPP, via its native MEP pathway (Figure 1) yields were typically insufficient. It is therefore widely recognized that pathway engineering is critical to ensuring a sufficient precursor pool for high-level heterologous isoprenoid synthesis in the host (Ward et al., 2018). Heterologous expression of the MVA pathway, found in eukaryotes including yeast and plants (Figure 1), has been found to dramatically improve isoprenoid synthesis in *E. coli*. Unlike the MEP pathway, it is not subject to feedback inhibition in the species allowing much greater precursor accumulation (Liu C.-L. et. al., 2019). However, MVA flux must be carefully balanced to minimize accumulation of toxic intermediates (Liu C.-L. et al., 2019).

Peralta-Yahya et al. (2011), expressed an eight gene MVA pathway to convert acetyl-CoA into FPP from a single plasmid in *E. coli* to provide a sufficient pool of the key sesquiterpene precursor. Co-expression of a second plasmid expressing a bisabolene synthase gene yielded 388 mg/L of the potential jet fuel feedstock. Through a series of metabolic engineering steps including codon optimization, modularization and promoter optimization, bisabolene titers were improved to 912 mg/L (Peralta-Yahya et al., 2011). An analogous method was recently applied for the synthesis of the monoterpenoid biojet fuel precursors, linalool and eucalyptol, in *E. coli* cell factories (Mendez-Perez et al., 2017). For eucalyptol biosynthesis, eight MVA pathway genes were initially expressed along with cineole synthase from a single plasmid, resulting in an optimal titer of 228 mg/L. Further metabolic engineering efforts to redistribute the pathway genes across two plasmids and balance MVA pathway flux allowed a three-fold improvement in eucalyptol titer to 653 mg/L (Mendez-Perez et al., 2017). Using a similar approach, linalool titers were also improved to a maximum titer of 505 mg/L (Mendez-Perez et al., 2017). This modularization approach was also later applied to produce pinene with an optimized titer of 32 mg/L (Sarria et al., 2014).

Liu et al. (2018), also employed the well established approach of expressing the MVA pathway and terpene synthase genes from separate hosts in *E. coli* for the production of three novel tricyclic sesquiterpenes, epi-isozizaene, pentalenene, and α-isocomene. Following effective promoter engineering maximum titers of 727.9, 780.3, and 77.5 mg/L were achieved in batch cultures of three engineered strains, respectively.

Recently, Wu et al. (2019), optimized production of another potential monoterpene jet fuel feedstock, limonene, in *E. coli* cell factories using a modularization approach. The limonene pathway was distributed between three modules, with the upstream module expressing MvaE and MvaS genes, responsible for the conversion of acetyl-CoA to MVA, originating from *Enterococcus faecalis*. The midstream module then expressed four genes responsible for the conversion of MVA to IPP/DMAPP originating from *Saccharomyces cerevisiae*. Finally, the downstream module contained the plant GPPS and limonene synthase genes. The up and midstream modules were carefully optimized through RBS and promoter engineering, respectively, with the optimal strain yielding 182 mg/L of limonene. Substituting the GGPP synthase gene for neryl diphosphate (NPP) synthase, which catalyzes the conversion of GPP to the alternative limonene substrate NPP, in the downstream module boosted limonene titers 2.9-fold to 695 mg/L. Finally, through fed batch cultivation of this optimized strain a maximum limonene titer of 1.29 g/L was achieved (Wu et al., 2019).

### S. cerevisiae

As in *E. coli*, pathway engineering is critical to ensuring a sufficient precursor pool for isoprenoid overproduction in *S. cerevisiae*. Peralta-Yahya et al. (2011), also engineered *S. cerevisiae* for bisabolene production. Genes encoding a truncated HMG-CoA reductase, FPP synthase and the global sterol pathway transcription regulator were overexpressed, whilst squalene synthase was downregulated to maximize FPP availability. Bisabolene synthesis was subsequently achieved through the expression a bisabolene synthase gene from a high-copy 2 μ plasmid, of the five bisabolene synthase genes tested, the optimal produced a maximum bisabolene titer of 994 mg/L (Peralta-Yahya et al., 2011).

Commercial scale biosynthesis of isoprenoids using microbial cell factories has been achieved using *S. cerevisiae*. Amyris first highlighted the potential of coupling metabolic engineering and synthetic biology during the semi-synthetic artemisinin project. Artemisinic acid, a key precursor to the antimalarial drug, artemisinin, was produced at commercial scale using a strategically engineered *S. cerevisiae* strain (Paddon and Keasling, 2014). Through the overexpression of all MVA pathway genes and use of a fed-batch cultivation strategy, impressive amorphadiene titers of around 40 g/L could be achieved (Westfall et al., 2012). This strain was later adapted for production of the advanced biofuel precursor, farnesene. However, as a pharmaceutical product, the value of artemisinin was around $150 per kg (Benjamin et al., 2016), whereas that of jet fuels is considerably lower. Industrially feasible synthesis of lower value commodities therefore requires dramatic production cost reductions. Meadows et al. (2016), identified the low ATP/oxygen ratio of *S. cerevisiae* as a key bottleneck. Through the introduction of heterologous reaction stoichiometries for acetyl-CoA, redox and sugar dissimilation, the conversion of sugar to farnesene was improved 25% while oxygen demand was reduced by 75% (Meadows et al., 2016). Despite alterations to fundamental cellular processes including glycolysis, the highly engineered strain thrived under harsh industrial conditions. Titers exceeding 130 g/L were achieved using unrefined cane syrup in 200,000 L fermenters (Meadows et al., 2016). Even with substantial progress and dramatic increases in productivity, however, production costs continue to limit farnesene to specialty chemical production.

### Yarrowia lipolytica

*Yarrowia lipolytica* is a non-conventional oleaginous yeast, which has gained substantial interest as a potential model industrial host for the production of lipid-based biofuels as it is able to accumulate large quantities of lipids, utilize low-cost renewable carbon sources, and tolerate wide range of pH and salinities (Liu Y. et al., 2019). Recently, *Y. lipolytica* was engineered for high level farnesene production (Liu Y. et al., 2019). Initially, three key MVA pathway genes, acetyl-CoA acetyltransferase, HMG-CoA synthase and HMG-CoA reductase were expressed from a linearized plasmid, which was subsequently randomly integrated into the genome of *Y. lipolytica*. A library of strains harboring these random integrations was constructed and screened for optimal MVA production. Two copies of a farnesene synthase-FPP synthase fusion along with additional copies of ERG12, ERG8, ERG19, IDI, and GPPS were subsequently integrated into the highest producing strain (1.96 g/L MVA). Cultivation of this strain resulted in a maximum farnesene titer of 1.7 g/L compared to 0.13 g/L for farnesene synthase alone. Finally, optimization of the fermentation conditions (pH, DO, and stirring speed), followed by high density fed batch fermentation led to an impressive farnesene titer of 25.55 g/L (Liu Y. et al., 2019).

## Microbial Second, Third, and Fourth Generation Isoprenoid Biofuel Synthesis

**FIGURE 3:**
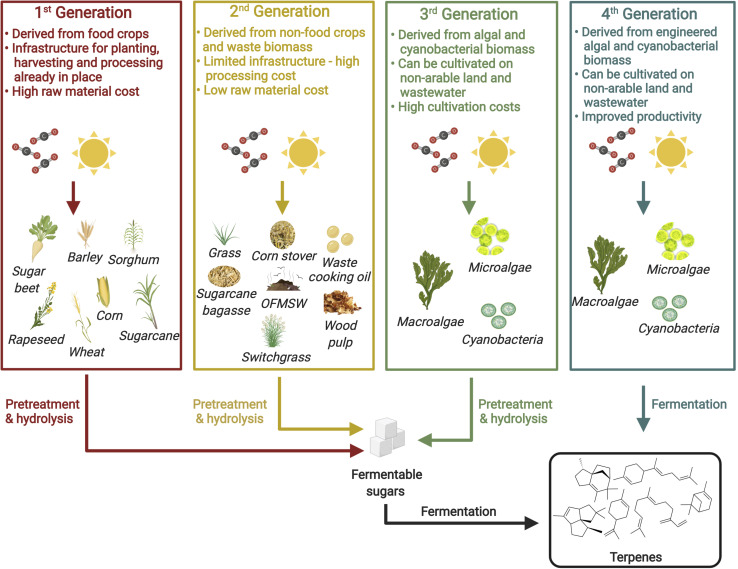
Potential feedstocks for biofuel production. Figure created with BioRender.com.

Although promising titers have been achieved for a number of isoprenoid biojet fuels across different microbial hosts as discussed in section “Microbial Production of Isoprenoid Jet Fuel Candidates From First Generation Feedstocks,” the use of first generation feedstocks remains ubiquitous. As such feedstocks are derived from edible crops, there widespread use results in increased food prices and threatens global food security (Mohr and Raman, 2013). The sustainable industrialization of isoprenoid based renewable jet fuels therefore relies on the use of second, third or fourth generation feedstocks. Such feedstocks are not derived from edible crops and do not compete with food crops for arable land. Figure 3 provides a summary of potential feedstocks for biofuel production and their key features. In this section, recent progress toward the production of biofuels from non-first generation feedstocks is presented.

### Second Generation Feedstocks

#### Lignocellulosic Biomass

Lignocellulosic biomass is the most abundant material on the planet for the production of low-cost commodities such as biofuels (Minty and Lin, 2015). Lignocellulose is comprised of the carbohydrate polymers cellulose and hemicellulose and the aromatic polymer lignin (Abdel-Hamid et al., 2013). In order to be metabolized by microbes the complex polymers must be hydrolyzed to release monomeric sugars. A major challenge in their use as feedstocks is their high xylose content, a pentose sugar which cannot naturally be metabolized by widely used microbial hosts such as *E. coli* and *S. cerevisiae*. In addition, pre-treatment leads to the release inhibitory compounds, which can greatly hinder cell viability and productivity. These include phenolic compounds (ferulic acid, *p*-coumaric acid), furan derivatives (furfural, 5-hydroxymethylfurfural) and small organic acids (levulinic acid, formic acid, and acetic acid) (Wang S. et al., 2017; Navarrete et al., 2020).

The non-conventional carotenogenic yeast *Rhodosporidium toruloides*, is able to metabolize both xylose and glucose simultaneously (Zhuang et al., 2019). *R. toruloides* also has the unique ability to metabolize a range of lignin associated aromatics including *p*-coumaric acid, ferulic acid, vanilic acid, and benzoic acid (Yaegashi et al., 2017). Such compounds inhibit the growth and productivity of *S. cerevisiae* (Larsson et al., 2000) and *E. coli*. As a result, this yeast is a promising host for the production of isoprenoids from lignocellulosic feedstocks, eliminating the need for extensive metabolic engineering to improve tolerance or expensive pre-treatment steps when using lignocellulosic feedstocks. As a result, the development of efficient metabolic engineering tools for this host is of growing research interest (Park et al., 2018; Nora et al., 2019).

Lignocellulosic corn stover waste was recently employed as a feedstock for cineole biosynthesis using an engineered *R. toruloides* strain. The heterologous genes were randomly integrated via NHEJ using an *Agrobacterium tumefaciens* mediated transformation method (Zhuang et al., 2019). The corn stover was first deacylated in dilute alkali, mechanically refined and enzymatically hydrolyzed to release high concentrations of monomeric (glucose and xylose) sugars. Following growth on this medium the engineered strain produced a maximum cineole titer of 34.6 mg/L (Zhuang et al., 2019). Although titers achieved with *R. toruloides* to date are lower than those achieved with the model yeast *S. cerevisiae*, several researchers have developed CRISPR-Cas9 based genome editing methods for *R. toruloides*, offering increased integration efficiency and targeted multiplex genome editing (Jiao et al., 2019; Otoupal et al., 2019; Schultz et al., 2019). The application of such tools and development of synthetic biology standards (Beal et al., 2020) to construct heterologous pathways for isoprenoid jet fuel candidate biosynthesis has the potential to boost titers from lignocellulose derived feedstocks significantly, thereby improving the economic feasibility of microbial biojet fuel production.

#### Glycerol

As a major waste product of biodiesel production, crude glycerol is a promising low-cost, second generation feedstock. In a recent study by You et al. (2017), the precursor to the approved jet fuel, farnesane, was produced from glycerol by an engineered *E. coli* strain. Although, encouraging titers of 8.7 g/L were achieved with pure glycerol, lower titers were achieved in subsequent experiments using crude glycerol. This was attributed to the presence of inhibitory compounds, indicating pre-treatment may be required to maximize productivity on crude glycerol (You et al., 2017). Glycerol was also shown to be preferred carbon source for limonene production by an engineered *E. coli* strain recently (Rolf et al., 2020). An impressive final limonene titer of 3.6 g/L was achieved in a glycerol limited fed-batch culture of the strain. *E. coli* has also been engineered for the production of the bicyclic monoterpene sabinene by Zhang et al. (2014). Process optimization studies using the engineered strain revealed the substitution of glucose with glycerol as the carbon source improved sabinene titers two-fold through fed-batch cultivation.

Cao et al. (2016) recently engineered a heterologous limonene pathway in *Y. lipolytica*. Through the overexpression of HMG1 along with codon optimized NPP and limonene synthase genes, followed by medium optimization, a maximum limonene titer of 23.6 mg/L was achieved from glucose. In a subsequent study by the group, an additional copy of limonene synthase was added to enhance limonene yields (Cheng et al., 2019). Of a range of carbon sources tested, interestingly glycerol was the optimal for limonene biosynthesis, with a maximum titer of 165.3 mg/L achieved in fed-batch fermentation (Cheng et al., 2019).

#### Waste Cooking Oil

Around 29 million tons of waste cooking oil are generated globally each year (Katre et al., 2017). As a single liter of waste cooking oil can pollute up to 500,000 liters of water (Panadare and Rathod, 2015), its safe disposal is a major environmental challenge. One potential solution, which has gained substantial interest, is the application of waste cooking oil as a feedstock for biodiesel production (Kulkarni and Dalai, 2006; Katre et al., 2017). A number of methods have been developed for the transesterification of the oil to yield biodiesel including alkali, acid and enzymatic transesterification. Alkali-catalyzed transesterification methods are widely used for the conversion of vegetable oil into biodiesel as they are considerably faster than acid catalyzed reactions. Such methods are hindered by the high levels of free fatty acids in waste cooking oil, however, which react with the catalyst forming soap. This prevents glycerol separation and dramatically reduces ester yields (Kulkarni and Dalai, 2006; Katre et al., 2017). Enzyme-catalyzed reactions can be conducted at lower temperatures with reduced pretreatment requirements and are insensitive to free fatty acids, however, the high costs associated with the required enzymes hider commercialization (Kulkarni and Dalai, 2006). In addition, the enzymes may be inactivated by methanol, the most widely used acyl acceptor for transesterification (Kulkarni and Dalai, 2006; Norjannah et al., 2016). As a result of such limitations and the vast quantities of waste cooking oil generated, alternative uses are desirable (Panadare and Rathod, 2015).

As a number of microorganisms are capable of utilizing waste cooking oils as a carbon source, they are gaining interest as an alternative feedstock for the production of heterologous products (Panadare and Rathod, 2015). Pang et al. (2019) recently capitalized on this ability in the non-conventional yeast *Y. lipolytica*. Using an engineered *Y. lipolytica* strain, the researchers successfully produced limonene from waste cooking oil. However, the resulting D-limonene and L-limonene titers were low at 2.5 and 2.7 mg/L, respectively, using 70% waste cooking oil as the carbon source. Despite this, titers obtained using cooking oil were over 10% higher than those using glucose indicating titers may be improved through MVA pathway optimization in the strain. Recently, Zhao et al. (2020) overexpressed ten genes of the MVA pathway in *Y. lipolytica* to enhance heterologous bisabolene synthesis. Expression of a heterologous efflux pump and supplementation of the medium with additional Mg^2+^ had a positive effect on productivity through reducing toxicity and increasing enzyme co-factor availability, respectively. Using waste cooking oil as a carbon source the optimal engineered strain achieved a maximum bisabolene titer of 973 mg/L (Zhao et al., 2020). A combination of waste cooking oil (carbon source) and corn steep liquor (sugar, nitrogen, amino acids, and vitamins source) has also been employed as a feedstock for the production of carotene by the fungus, *Blakeslea trisporai* (Nanou et al., 2017). Cultivation in a bubble column reactor resulted in a maximum carotene titer of 980 mg/L. The gram-negative bacterium, *Pseudomonas aeruginosa*, has also been cultivated on a number of waste oils for the production of biosurfactants (Pérez-Armendáriz et al., 2019). An impressive rhamnolipid titer of 3.6 g/L was achieved when the strain was grown on media containing just waste canola oil and a combination of four salts (Pérez-Armendáriz et al., 2019). Although research into the microbial conversion of waste cooking oil into isoprenoid products is in its infancy, its low cost and high abundance renders it an attractive feedstock for the sustainable production of advanced biojet fuels.

### Third and Fourth Generation Feedstocks

Third and fourth generation feedstocks, which are derived from photosynthetic organisms including algae and cyanobacteria, are of growing interest for biofuel production as they exhibit higher rates of photosynthesis and growth than terrestrial plants (Dutta et al., 2014). As phototrophs, the application of such hosts would also eliminate the need for costly carbon sources. Furthermore, many microalgae and cyanobacterial species can be grown in wastewater, where they would utilize harmful nitrogen and phosphorus for growth, resulting in effective bioremediation and a reduced reliance on freshwater (Abedi et al., 2019; Hernández-García et al., 2019). In the case of third generation terpene biojet fuel production the biomass would provide the carbon source for an engineered host (Figure 3). Microalgae, macroalgae and cyanobacterial biomass has been employed for the production of third generation bioethanol (de Farias Silva and Bertucco, 2016; del Río et al., 2019). Following biomass pretreatment and hydrolysis, to degrade the cell wall and release fermentable sugars, the resulting sugar was then fermented by *S. cerevisiae* yielding bioethanol. Fourth generation terpene biojet fuel production on the other hand, would involve an engineered algae or cyanobacterium capable of synthesizing the terpene of interest directly (Figure 3). As the primary carbon source of such organisms is CO_2_, the environmental impact of fourth generation biofuels is likely to be reduced dramatically compared to bacterial or yeast hosts. The cyanobacteria, *Synechocystis* sp., is a non-conventional host under investigation as a potential host for fourth generation advanced biofuel production. *Synechocystis* sp. was recently engineered for the production of bisabolene, however, even after extensive metabolic engineering efforts, the rate of photosynthesis remained limiting and 36 days of growth were required to produce just 22 mg/L of bisabolene (Sebesta and Peebles, 2020). The species has also been engineered for the production of the monoterpene, limonene, with a maximum titer of 6.7 mg/L achieved following 7 days of cultivation (Lin et al., 2017).

Other microorganisms with potential as third and fourth generation feedstocks include acetogens such as *Clostridium autoethanogenum*, which are capable of producing ethanol from syngas and industrial waste gasses (Heffernan et al., 2020). Such organisms provide a means of capturing and recycling carbon from waste gases that would otherwise be released into the atmosphere and contribute to environmental pollution. Lanzatech are striving to capitalize on the potential of the species to develop wide-ranging sustainable fuels and chemicals (LanzaTech, 2017). They recently commercialized an innovative waste gas to bioethanol process using *C. autoethanogenum* (Heffernan et al., 2020). The company have also successfully engineered the species for the production of farnesene from a carbon monoxide containing waste gas (Yiting Chen et al., 2015).

A summary of each of the discussed fuel candidates along with the microbial host and feedstock is provided in Table 3.

**TABLE 3:** Summary of recent progress in microbial biosynthesis of isoprenoid-derived advanced jet fuel candidates.

Microbial host	Feedstock	Terpene	Potential biojet fuel blendstock	Titer (mg/L)	Scale	Operation	References
*E. coli*	Glucose	Bisabolene	Bisabolane	912	50 mL Flask	Batch	Peralta-Yahya et al. (2011)
		Linalool	RJ-4	505	Flask	Batch	Mendez-Perez et al. (2017)
		Eucalyptol	Limonane	653	Flask	Batch	Mendez-Perez et al. (2017)
		Pinene	Pinene dimers/pinane	32	5 mL Flask	Batch	Sarria et al. (2014)
		Epi-isozizaene	Epi-isozizaane	728	4 L Flask	Batch	Liu et al. (2018)
		Pentalenene	Pentalenane	780	2.5 L Flask	Batch	Liu et al. (2018)
		α-Isocomene	α-Isocomane	78	Flask	Batch	Liu et al. (2018)
		Limonene	Limonene/limonane	1,290	Flask	Fed-batch	Wu et al. (2019)
	Pure glycerol	Farnesene	Farnesane	8740	7 L Bioreactor	Batch	You et al. (2017)
		Limonene	Limonene/limonane	3,600	3.1 L Bioreactor	Fed-batch	Rolf et al. (2020)
		Sabinene	Sabinane	2,650	5 L Bioreactor	Fed-batch	Zhang et al. (2014)
		Myrcene	2,6-dimethyloctane	58	50 mL Flask	Batch	Kim et al. (2015)
	Crude glycerol	Farnesene	Farnesane	2,830	7 L Bioreactor	Batch	You et al. (2017)
*S. cerevisiae*	1.8% galactose/0.2% glucose	Bisabolene	Bisabolane	994	5 mL Flask	Batch	Peralta-Yahya et al. (2011)
		Pentalenene	Pentalenane	344	5 mL Flask	Batch	Liu et al. (2018)
	Unrefined cane syrup	Farnesene	Farnesane	130,000	200,000 L Bioreactor	Fed-batch	Meadows et al. (2016)
*Y. lipolytica*	Glucose	Farnesene	Farnesane	2,555	1 L Bioreactor	Batch	Liu Y. et al. (2019)
	Glycerol	Limonene	Limonene/limonane	165	1.5 L Bioreactor	Fed-batch	Cheng et al. (2019)
	Waste cooking oil	Limonene	Limonene/limonane	2.5	Flask	Batch	Pang et al. (2019)
		Bisabolene	Bisabolane	973	250 mL Flask	Batch	Zhao et al. (2020)
*R. toruloides*	Corn stover hydrolysate	Eucalyptol	Limonane	35	2 L Bioreactor	Batch	Zhuang et al. (2019)
		Bisabolene	Bisabolane	680	2 L Bioreactor	Fed-batch	Yaegashi et al. (2017)
*Synechocystis* sp.	CO_2_	Bisabolene	Bisabolane	22	3 L Photobioreactor	Batch	Sebesta and Peebles (2020)
		Limonene	Limonene/limonane	6.7	250 mL Flask	Batch	Lin et al. (2017)

## Bioprocessing Strategies for Biojet Fuel Production From Lignocellulosic Feedstocks

**FIGURE 4:**
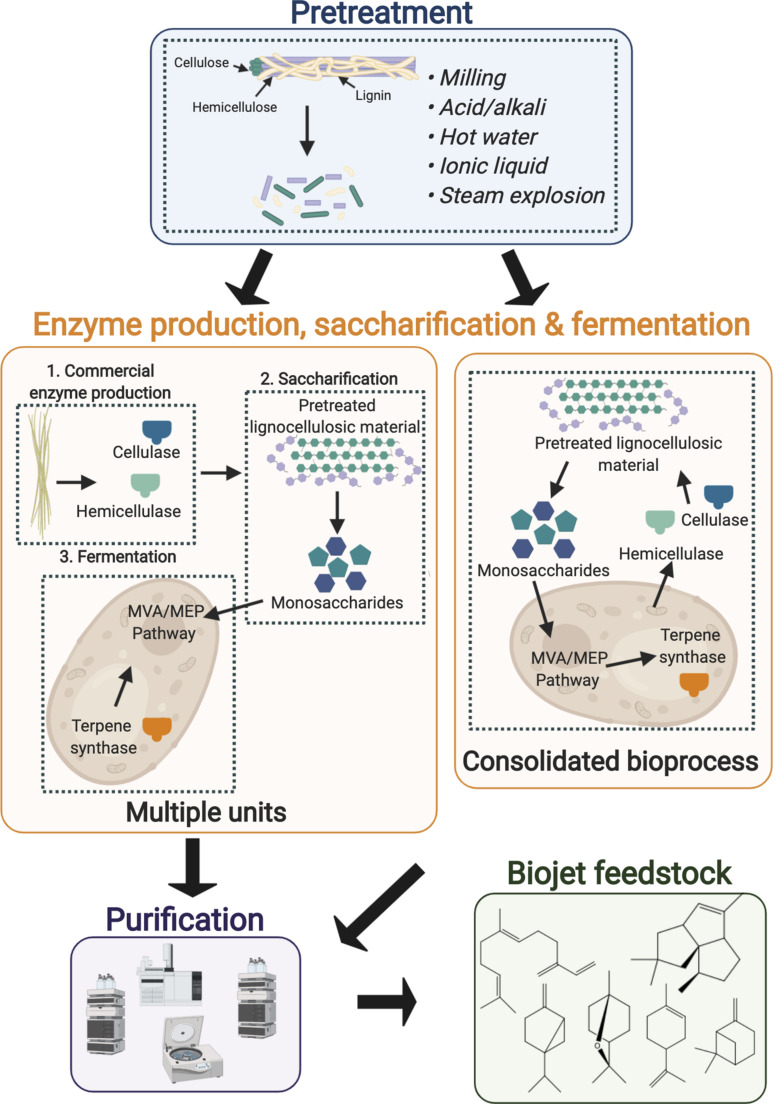
Bioprocessing methods for the conversion of lignocellulosic biomass to advanced jet fuel feedstocks. Firstly the raw biomass is pretreated to reduce crystallinity and lignin content and to increase the surface area prior to enzymatic hydrolysis. The pretreated biomass then undergoes saccharification to release the monomeric sugars, which can be converted into the product of interest by the microbial host. The enzyme production, saccharification and fermentation steps may be performed separately (multiple units) or consolidated into a single unit (consolidated bioprocess). Dashed lines represent single unit operations. Figure created with BioRender.com.

As over half of the carbon in the biosphere is in the form of cellulose, lignocellulosic biomass is the most abundant, low cost feedstock available for commercial microbial biofuel production (Minty and Lin, 2015). However, the high costs associated with its pre-treatment and enzymatic hydrolysis and the inhibitory compounds generated during these steps currently hinders the economic feasibility of their widespread use at commercial scale. Firstly, the biomass is typically subjected to physical, chemical, biological or a combination of the latter pre-treatment steps to separate and solubilize the complex components for improved digestibility (Minty and Lin, 2015; Chen et al., 2017). The pretreated biomass then undergoes saccharification, in which the cellulose and hemicellulose are enzymatically hydrolyzed releasing monomeric sugars. The monomeric sugars can then be metabolized by the engineered host to produce the biojet fuel feedstock of interest (Figure 4). Using a traditional approach each of the aforementioned steps are performed in a separate unit operation (multiple units, Figure 4) resulting in high capital and operational costs thus hindering the economic feasibility of low value commodity production from lignocellulosic feedstocks. Each gram of crystalline cellulose requires 30–50 mg of commercial enzymes (Ali et al., 2016), such high loading requirements and the slow rate of digestion are particular bottlenecks. CBP is a promising alternative approach, in which cellulase production, enzymatic saccharification and microbial fermentation are consolidated in a single unit operation (consolidated bioprocess, Figure 4) (Minty and Lin, 2015). Such approaches could improve the economic feasibility of jet biofuel synthesis through reduced feedstock processing complexity, lower energy requirements and increased conversion efficiency compared to multiple units (Levin et al., 2015).

There are three potential approaches to developing a CBP for the microbial conversion of lignocellulosic biomass to advanced jet biofuels as follows:

1. Engineer a naturally highly efficient cellulolytic microbe to produce the desired product
2. Engineer a microbe with a high product titer to express hydrolytic enzymes to efficiently solubilize the lignocellulosic substrate and to consume both hexose and pentose sugars
3. Engineer a microbial consortia allowing labor to be divided across multiple microorganisms

### Engineering Cellulolytic Microbial Hosts for Heterologous Terpene Biosynthesis

Filamentous fungi such as *Trichoderma* are the largest producers of lignocellulolytic enzymes in industry (Liu and Qu, 2019) and produce a wide range of volatile organic compounds (VOC) as secondary metabolites (Stoppacher et al., 2010; Contreras-Cornejo et al., 2014). A significant proportion of these VOCs are terpenes, mostly monoterpenes and sesquiterpenes. For example, *Trichoderma atroviride* was found to naturally produce a number of the aforementioned advanced biojet fuel candidates including β-farnesene, α-farnesene, and β-bisabolene (Stoppacher et al., 2010). Similarly, a number of endophytic fungi have also been demonstrated to be promising CBP hosts for the production of advanced biofuels (Wu W. et al., 2017). Wu W. et al. (2017), successfully cultivated four endophytic fungi using a number of lignocellulosic materials as the sole carbon sources including switchgrass, corn stover and eucalyptus mill powder. The fungi naturally accumulated a wide range of VOC as secondary metabolites, of which a significant proportion (up to 63%) were terpenes. The majority of the produced terpenes were monoterpenes or sesquiterpenes, and included some known biofuel candidates such as α-pinene, β-pinene, limonene and cineole (Wu W. et al., 2017). In another endophytic fungus, *Annulohypoxylon* sp., the advanced jet fuel precursor, eucalyptol, comprised up to 94% of the VOCs produced depending on the ligncellulosic waste used as the carbon source (Wang K.Y. et al., 2017). Although relatively low titers will necessitate significant metabolic engineering to reach commercial production levels, the natural ability of certain fungi to both metabolize lignocellulosic materials and synthesize advanced biojet fuel feedstocks renders them excellent candidate CBP hosts.

Recently, Pang et al. (2017) isolated a novel cellulolytic *E. coli* strain from bovine rumen. With naturally evolved extracellular cellulase ethanol production, the strain effectively metabolized corn straw, representing a promising potential CBP host (Pang et al., 2017). The thermophilic bacteria, *C. thermocellumas*, is another promising CBP host candidate due to its remarkably high enzymatic hydrolysis efficiency (Akinosho et al., 2014). The species grows well at temperatures between 50 and 68°C, minimizing contamination risks. However, as it is unable to metabolize pentose sugars, further metabolic engineering would likely be necessary to maximize substrate conversion.

The non-conventional yeast *Spathaspora passalidarum* is capable of fermenting xylose faster than glucose and can co-metabolize glucose and xylose under both aerobic and anaerobic conditions (Chen et al., 2015; Morales et al., 2017). However, the lack of genetic engineering tools and relatively poor tolerance of this strain to lignin associated inhibitory compounds has hindered microbial cell factory development to date. Yaegashi et al. (2017) recently demonstrated a novel single-unit lignocellulose pre-treatment, saccharification, and fermentation process for heterologous bisabolene biosynthesis using the non-conventional yeast, *R. toruloides*. Initially the corn stover feedstock was pre-treated using the biocompatible ionic liquid, choline α-ketoglutarate, to reduce cellulose crystallinity and lignin content. The pH of the resulting mixture was reduced to five for the subsequent enzyme hydrolysis step, in which cellulase and hemicellulase were added to release the monomeric sugars. The result of this step was a medium containing 17.1 g/L glucose, 9.1 g/L xylose, and 383 mg/L of *p*-coumaric acid (Yaegashi et al., 2017). Subsequent cultivation of the engineered *R. toruloides* strain in this medium resulted in a final bisabolene titer of 261 mg/L. Interestingly the titer achieved using this hydrolysate was over two-fold higher than in the synthetic control medium containing equal quantities of glucose, xylose, and *p*-coumaric acid (Yaegashi et al., 2017). Although this represents substantial progress toward the development of a CBP through combining pre-treatment, saccharification and fermentation into a single unit operation, the process relied on costly commercial enzymes for substrate hydrolysis.

### Engineering Model Hosts to Produce Cellulases and Advanced Biofuels

Recently Amyris undertook an ambitious project in collaboration with Renmatix and Total to engineer their industrial farnesene overproducing *S. cerevisiae* cell factories to grow on lignocellulosic feedstocks with the ultimate goal of reducing production costs to $2 per liter (Mitrovich and Amyris, 2019). This involved extensive metabolic engineering of the *S. cerevisiae* cell factories to facilitate xylose catabolism along with resistance toward growth inhibitors. However, even following an extensive engineering effort, the predicted minimum production cost was $4–5/L resulting in premature termination of the project.

A number of recombinant *S. cerevisiae* strains with the ability to metabolize xylose have been successfully constructed. *S. cerevisiae* was recently engineered for the production of a carotenoid, lycopene, from a mixed glucose-xylose substrate (Su et al., 2020). Through engineering heterologous pathways for xylose consumption and lycopene synthesis a maximum titer of 903 mg/L was achieved in fed-batch culture on the mixed substrate (Su et al., 2020). However, preference for glucose and slow and repressive consumption of xylose during co-consumption of the two sugars remain major bottlenecks (Chen et al., 2015). In addition, such strains typically still rely on the use of commercial enzymes for saccharification.

*Saccharomyces cerevisiae* was also recently engineered for the heterologous expression of α-amylase and glucoamylase to develop a consolidated enzyme production, saccharification and glucose fermentation process using raw starch as a substrate (Cripwell et al., 2019). The resulting strain achieved yields in excess of 80% of the theoretical maximum.

### Division of Labor

#### Microbial Consortia

Microbial consortia are ubiquitous in nature and play important roles in nutrient recycling and maintaining the health of humans, animals and plants (McCarty and Ledesma-Amaro, 2019). Such organisms are capable of performing tasks too complex for any single organism to complete alone through the strategic division of labor. Many natural consortia are innately capable of converting lignocellulosic biomass to a plethora of natural products, however, the predominant products are organic acids, CO_2_ and methane which are not suitable for advanced liquid biofuel applications (Zuroff et al., 2013). The assembly of effective synthetic microbial consortia for the pre-treatment, saccharification and fermentation of lignocellulosic biomass into terpene fuels could help alleviate some of the major bottlenecks associated with monocultures. A co-culture of the thermophilic bacteria, *C. thermocellum* and *C. thermolacticum*, showed improved ethanol production compared to mono-cultures of the strains (Xu and Tschirner, 2011). This was attributed to combining the higher enzymatic hydrolysis efficiency of *C. thermocellum* and ability of *C. thermolacticum* to metabolize all of the hydrolysis products including xylose. The ethanol yield from cellulose of resulting co-culture was 75% of the theoretical maximum (Xu and Tschirner, 2011). An *E. coli–E. coli* co-culture has been engineered for the conversion of switchgrass biomass into the monoterpene, pinene (Bokinsky et al., 2011). The plant biomass was first pre-treated through dissolution in an ionic liquid to reduce crystallinity and lignin content thereby improving digestibility. Two engineered *E. coli* strains were subsequently employed for pinene synthesis, the first harboring genes for xylan hydrolysis and the second for pinene synthesis. The resulting co-culture yielded 1.7 ± 0.6 mg/L of pinene (Bokinsky et al., 2011). Although this yield is relatively low, a large proportion of the switch grass was not metabolized indicating the process could be improved through further metabolic engineering.

The construction of stable synthetic consortia is, however, limited by a lack of understanding of how most species interact (Peng et al., 2016). In addition, those strains engineered for high level terpene production are highly domesticated to thrive independently under laboratory conditions, which may hinder their ability to form robust co-cultures with cellulolytic microorganisms.

#### Division of Labor Across Separate Units

One potential solution recently proposed by Navarrete et al. (2020) involves the use of sequential bioreactors, in the first feedstock degradation occurs, whilst in the second biochemical production is performed. This two stage approach has been demonstrated for lipid biosynthesis (Xu et al., 2015). The first stage involved the anaerobic digestion of macroalgae biomass (30 g/L) by microbes obtained from anaerobic digester sludge from a wastewater treatment plant. The sludge was heat-treated to inactivate methanogens prior to use as the inoculum for the macroalgae fermentation. This produced a mixture of volatile fatty acids (VFAs) (acetic acid 8.24 g/L, propionic acid 1.11 g/L, and butyric acid 0.45 g/L), which subsequently provided the carbon source for a second reactor containing either *Y. lipolytica*, *Cryptococcus curvatus*, or *Trichosporon dermatis*. Of the yeast species investigated, *Cr. curvatus* and *T. dermatis* successfully consumed all of the acetic acid during 60 h cultivations and produced 1.3 and 1.3 g/L of lipids, respectively (Xu et al., 2015).

Ruminants are an excellent example of a naturally evolved CBP. The rumen microbiome is a highly evolved symbiotic community of bacteria, fungi, protozoa, bacteriophages and methanogens capable of exceptionally efficient lignocellulose degradation (Ribeiro et al., 2016). This consortia break down the lignocellulosic biomass releasing VFA, mainly acetic, propionic and butyric acids, which are subsequently used as the primary source of carbon by the animal to produce valuable products (i.e., meat and milk). As a result, the rumen is of growing interest as a potential source of CBP hosts and lignocellulose degrading enzymes. Predominant species of ruminal bacteria include *Ruminococcus albus*, *Ru. flavefaciens*, and *Fibrobacter succinogenes* (Weimer et al., 2009; Sharma et al., 2019). The rumen bacteria, *Ru. albus* has also been employed for the depolymerization of sorghum and bagasse wastes to release fermentable sugars for the subsequent conversion to ethanol by *S. cerevisiae* (Mutalik et al., 2012). Fermentation of the filtered hydrolyzed wastes resulted in ethanol titers of 17.4 and 19.8 g/L, respectively (Mutalik et al., 2012).

**FIGURE 5:**
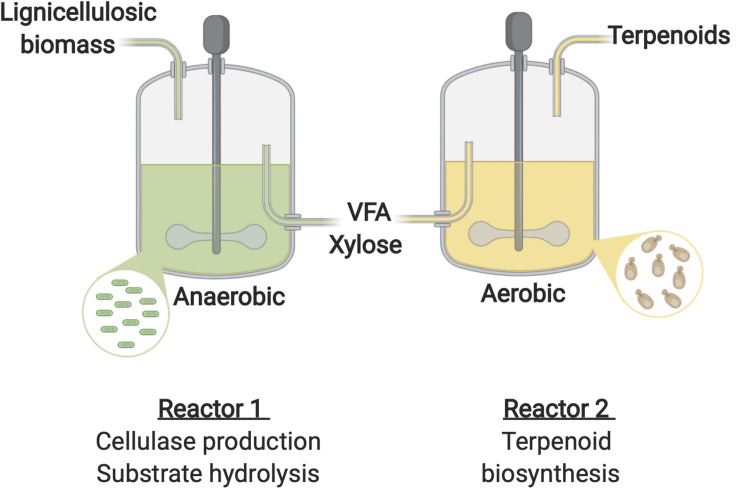
Proposed sequential bioprocessing strategy for terpene production from lignocellulosic biomass. Figure created with BioRender.com.

A number of oleaginous yeasts including *R. toruloides* (Huang et al., 2016), *Y. lipolytica* (Llamas et al., 2020), *Cr. curvatus* (Liu et al., 2017), and *T. dermatis* (Xu et al., 2015) have been employed for the production of lipids using VFA as the sole carbon source. However, optimal conditions vastly different with rumen bacteria performing optimally under anaerobic conditions at temperatures between 37 and 39°C (Sari et al., 2017) and circumneutral pH (Wu C. et al., 2017), while growth of the oleaginous yeast requires aeration and is typically optimal under acidic conditions at around 30°C (Dai et al., 2019; Hackenschmidt et al., 2019). In addition, at high concentrations lignocellulosic hydrolysate components such as acetic acid (∼40 g/L; Huang et al., 2016), formic acid (2 g/L; Zhao et al., 2012). and vanillin (1.5 g/L; Zhao et al., 2012) have an inhibitory effect on *R. toruloides.* Such discrepancies would likely hinder effective simultaneous enzyme production, saccharification and fermentation to advanced biojet fuels by a consortia of this nature. A sequential bioprocess could therefore be a promising approach for the production of advanced biojet fuels from lignocellulosic biomass using such microbes (Figure 5). In the first reactor a cellulolytic bacterium or consortia would be employed for efficient lignocellulose degradation. *F. succinogenes*, is a rumen cellulolytic bacterium capable of robust and efficient lignocellulosic biomass degradation (Raut et al., 2019). It ferments the resulting cellobiose and glucose products generating VFAs. Interestingly the species possesses xylanase activity and is therefore capable of converting xylan into xylose despite being unable to metabolize the sugar (Forano et al., 2008; Suen et al., 2011). *F. succinogenes* could therefore be a promising microorganism for this stage. The resulting product comprised of VFA and xylose would provide the carbon source for a yeast species such as *Cr. curvatus* or *R. toruloides* capable of efficient xylose and VFA metabolism and engineered for terpene overproduction in a second reactor. This approach would allow the optimal conditions for each microorganism to be maintained in their respective reactors thereby maximizing productivity. In addition, a fed-batch strategy could be applied for the terpene synthesis stage to prevent VFA and lignin aromatics accumulating to inhibitory concentrations.

Although all of the methods discussed in this section, demonstrate potential for improving the sustainability of microbial isoprenoid biojet fuel production, a holistic approach is likely to be necessary to ensure economic viability at industrial scale. Through the coupling of non-conventional hosts, which are naturally capable of growing on abundant, low cost feedstocks and engineered to overproduce the terpene fuel of interest, with strategic bioprocessing methods such as those discussed here, critical commercial bottlenecks such as high raw material and/or pre-treatment costs could be effectively alleviated. Both economic and environmental sustainability would be improved dramatically, allowing the feasibility of industrial scale isoprenoid derived biojet fuel production to be enhanced.

## Conclusion

This review focused on potential isoprenoid derived advanced biojet fuel candidates and progress toward their microbial production. Such compounds have properties similar to traditional aviation fuels and are compatible with existing infrastructure. Although extensive progress toward microbial production of isoprenoid derived advanced biofuels has been made recently, the use of first generation feedstocks remains ubiquitous. The development of an economically and environmentally sustainable bioprocess is likely to require the use of waste derived feedstocks. Examples including crude glycerol, waste cooking oil, and lignocellulosic biomass, which have recently been demonstrated as effective substrates for isoprenoid biosynthesis were considered in this review. Emphasis was made on the most abundant renewable feedstock, lignocellulosic biomass and major bottlenecks, which have hindered its widespread application to date. The high costs associated with the pretreatment and saccharification of this complex feedstock limits its economic feasibility for low-value commodity production. In addition, lignocellulosic biomass is comprised of up to 40% pentose sugars, which cannot be naturally metabolized by *S. cerevisiae* or *E. coli* and the lignin associated compounds released during lignocellulosic biomass pretreatment inhibit growth of these model hosts. The potential of a number of innovative bioprocessing methods such as CBP and sequential bioreactors to alleviate such bottlenecks were evaluated in this review. Opportunities to capitalize on the robust and efficient lignocellulosic degradation capability of novel cellulolytic bacteria and filamentous fungi were investigated. Non-conventional yeasts such as, *R. toruloides*, with the ability to metabolize xylose faster than glucose and both tolerate and metabolize lignin associated compounds were considered as alternative hosts for the conversion of lignocellulose to advanced jet fuels. The development of an effective bioprocess for high level advanced biojet fuel production from lignocellulosic feedstocks using traditional model hosts has proven unsuccessful to date. Despite this, through the adoption of novel and synergistic approaches such as those discussed in this review, there is great potential to improve the sustainability of microbial isoprenoid biojet fuel production. Future research should focus on improving the economic viability and productivity of second, third or fourth generation isoprenoid biojet fuels, as such fuels promise substantial progress toward several of the UN’s Sustainable Development Goals and IATA’s commitment to reducing CO_2_ emissions by 50% by 2050.

## Author Contributions

All authors listed have made a substantial, direct and intellectual contribution to the work, and approved it for publication.

## Conflict of Interest

The authors declare that the research was conducted in the absence of any commercial or financial relationships that could be construed as a potential conflict of interest.
